# Short term sequelae of preeclampsia: a single center cohort study

**DOI:** 10.1186/s12884-018-1796-z

**Published:** 2018-05-21

**Authors:** Michael Girsberger, Catherine Muff, Irene Hösli, Michael Jan Dickenmann

**Affiliations:** 1 Clinic for Transplantation Immunology and Nephrology, University Hospital Basel, Petersgraben 4, 4031 Basel, Switzerland; 2 Department of Gynaecology and Obstetrics, University Hospital Basel, Spitalstrasse 21, 4031 Basel, Switzerland

**Keywords:** Preeclampsia, Follow-up, Sequelae

## Abstract

**Background:**

Data on the prevalence of persistent symptoms in the first year after preeclampsia are limited. Furthermore, possible risk factors for these sequelae are poorly defined. We investigated kidney function, blood pressure, proteinuria and urine sediment in women with preeclampsia 6 months after delivery with secondary analysis for possible associated clinical characteristics.

**Methods:**

From January 2007 to July 2014 all women with preeclampsia and 6-months follow up at the University Hospital Basel were analyzed. Preeclampsia was defined as new onset of hypertension (≥140/90 mmHg) and either proteinuria or signs of end-organ dysfunction. Hypertension was defined as a blood pressure ≥ 140/90 mmHg or the use of antihypertensive medication. Proteinuria was defined as a protein-to-creatinine ratio in a spot urine > 11 mg/mmol. Urine sediment was evaluated by a nephrologist. Secondary analyses were performed to investigate for possible parameters associated with persistent symptoms after preeclampsia.

**Results:**

Two hundred two women were included into the analysis. At a mean time of follow up of 172 days (+/− 39.6) after delivery, mean blood pressure was 124/76 mmHg (+/− 14/11, range 116–182/63–110) and the mean serum-creatinine was 61.8 μmol/l (33–105 μmol/l) (normal < 110 μmol/l). Mean estimated glomerular filtration rate using CKD-EPI was 110.7 mml/min/1.73m^2^ (range 59.7–142.4 mml/min/1.73m^2^) (normal > 60 mml/min/1.73m^2^). 20.3% (41/202) had a blood pressure of 140/90 mmHg or higher (mean 143/89 mmHg) or were receiving antihypertensive medication (5.5%, 11/202). Proteinuria was present in 33.1% (66/199) (mean 27.5 mg/mmol). Proteinuria and hypertension was present in 8% (16/199). No active urine sediment (e.g. signs of glomerulonephritis) was observed. Age and gestational diabetes were associated with persistent proteinuria and severe preeclampsia with eGFR decline of ≥ 10 ml/min/1.73m^2^.

**Conclusion:**

Hypertension and proteinuria are common after 6 months underlining the importance of close follow up to identify those women who need further care.

## Background

Preeclampsia is a pregnancy related disease defined as new onset of hypertension and either proteinuria or signs of other end-organ dysfunction (e.g. hepatic abnormality, pulmonary edema, thrombocytopenia). Preeclampsia occurs in approximately 5% of pregnancies [1], and is therefore a frequent disorder complicating gestation. Apart from the high morbidity with sometimes even life-threatening implications for mother and child during the acute phase of the disease, there are also concerns about long-term sequelae. Studies in the past have shown an increased risk of kidney biopsy [2] indicating kidney disease or end-stage renal failure [3, 4]. There is also evidence of increased risk of chronic hypertension following an episode of preeclampsia [5–7].

To identify those with chronic hypertension or proteinuria after delivery, it is important to know the usual time of resolution of these symptoms. Guidelines state that blood pressure should normalize in the first 3 months [8] and referral is considered if hypertension or proteinuria persists after three to 6 months [9, 10]. However, there is data indicating that hypertension persists in almost 40% of patients after 3 months and still is present in 18% after 2 years [11]. In one study almost 29% of patients were hypertensive after 5 years, although sample size was small [12]. Therefore, the time to define chronicity of symptoms remains unclear. In regard to the few existing studies, we hypothesized that a significant part of patients still show sequelae of preeclampsia 6 months after pregnancy. The aim of the study was to determine the frequency of hypertension, proteinuria and eGFR (estimated glomerular filtration rate) decline 6 months after preeclampsia and to search for possible parameters associated with these endpoints.

## Methods

From January 2007 to July 2014 all women with preeclampsia at the University Hospital Basel referred to our nephrology clinic were included into the study. As by our hospital policy, all patients with preeclampsia are referred for nephrology follow-up after 6 months. Patients were closely followed by their family doctor or obstetrician and referred earlier if necessary. Preeclampsia was defined as new onset of hypertension (≥140/90 mmHg) after the 20th gestational week or worsening hypertension (defined as blood pressure values ≥20/10 mmHg higher than previously measured during pregnancy) in patients with pre-existing elevated blood pressure and either proteinuria or other signs of end-organ dysfunction. The gynaecology department of the University Hospital of Basel had been in the practice of defining preeclampsia without the proteinuria requirement, as was later confirmed by the ACOG 2013 guidelines [13]. Hypertension was defined as a blood pressure ≥ 140/90 mmHg or the use of antihypertensive medication. This definition was used for inclusion into the study as well as at follow up. The presence of severe hypertension (≥160/110 mmHg), acute renal failure or oliguria, eclamptic seizure, pulmonary lung oedema, signs of HELLP-Syndrome, hyperreflexia, severe headaches, visual disturbances or intra uterine growth retardation with or without pathological Doppler ultrasound resulted in a diagnosis of severe preeclampsia [14]. Acute kidney injury was defined as a rise in serum creatinine concentration of more than 50% from baseline during hospitalisation. The presence of pre-existing conditions like hypertension, diabetes mellitus or kidney disease was gathered from medical records. Proteinuria was defined as a protein-to-creatinine ratio in a spot urine > 11 mg/mmol [15]. Decline of kidney function was defined as a decrease in eGFR ≥10 ml/min/1.73m^2^. Urine sediments were evaluated by a staff nephrologist. For secondary analyses, a multivariate logistic regression model was applied. Univariate analysis was conducted in all variables with at least 15 observations and variables with a *p*-value < 0.2 were added to the model. Wilcoxon Mann Whitney test was used to compare medians due to skewed distribution of the baseline characteristics in the subgroups; chi-squared test was used to compare frequencies. Different denominators in the results section are due to missing data in a few patients.

## Results

Of the 225 women referred to our nephrology clinic, 23 were lost to follow-up (10%). Two hundred two were included in the analysis. The mean time of the follow up visit was 172 days (+/− 39.6) after delivery. Mean age of the 202 women was 32 years (18–45 years). 58.2% (117/201) were pregnant for the first time (primigravida). In 22.2% (43/194) of the patient’s preeclampsia occurred before 34 weeks of gestation. Severe preeclampsia was observed in 67% (132/197) and eclampsia as the most severe form was seen in 2% (4/198). 90.1% (181/201) had no pre-existing diseases before pregnancy (diabetes mellitus, chronic kidney disease or hypertension). Baseline characteristics are shown in Table 1.

**Table 1:** Baseline characteristics

Age mean (±SD)	32 (± 5.9)
Onset of preeclampsia (gestational week) mean (±SD)	36 + 3 (± 3.9 weeks)
Early Onset (< 34 weeks of gestation) (43/195)	22.1%
Nulliparous (150/199)	75.4%
Multiple pregnancy (twins, triplets) (27/202)	13.4%
In vitro fertilisation (13/202)	6.4%
Diabetes before pregnancy (4/201)	1.99%
Gestational diabetes (19/202)	9.4%
Previous hypertension (16/202)	7.9%
HELLP (41/198)	20.7%
Eclampsia (4/198)	2.0%
Severe preeclampsia (132/197)	67%
Acute kidney injury (17/197)	8.6%
Chronic kidney disease (4/201)	1.99%

**Fig. 1:**
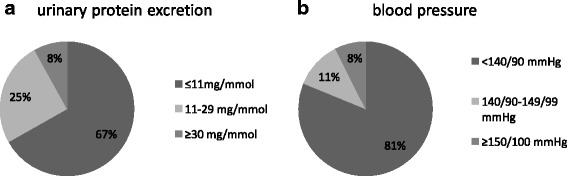
**a** and **b** Distribution of urinary protein excretion and blood pressure at follow-up

**Fig. 2:**
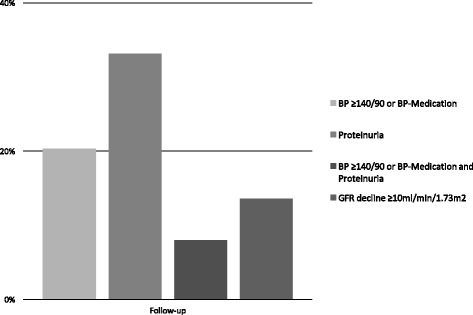
Prevalence of hypertension, proteinuria and eGFR decline at mean follow-up of 172 days (± 39.6) after delivery

Distribution of blood pressure and urinary protein excretion at follow-up are shown in Fig. 1a and b. The mean blood pressure at 6-months follow up was 124/76 mmHg (+/− 14/11, range 116–182/63–110) and the mean serum-creatinine was 61.8 μmol/l (33–105 μmol/l) (normal < 110 μmol/l). Mean estimated glomerular filtration rate (eGFR) using CKD-EPI (chronic kidney disease epidemiology collaboration) was 110.7 ml/min/1.73m^2^ (59.7–142.4 ml/min/1.73m^2^) (normal > 60 mml/min/1.73m^2^). 20.3% (41/202) had a blood pressure of 140/90 mmHg or higher (mean 143/89 mmHg) or received antihypertensive medication (5.5%, 11/202). Proteinuria was present in 33.1% (66/199) (mean 27.5 mg/mmol, range 12–261 mg/mmol). Proteinuria and hypertension were present in 8% (16/199) (Fig. 2). 54.3% (108/199) had none of the investigated sequelae at follow up. No active urine sediment (e.g. glomerular microhematuria, casts, signs of glomerulonephritis) was observed.

Baseline characteristics in women with sequelae at follow-up are shown in Tables 2, 3 and 4. Multivariate logistic regression analyses showed an association of age and gestational diabetes with proteinuria > 11 mg/mmol and proteinuria ≥30 mg/mmol at follow-up, respectively. Additionally, severe preeclampsia was associated with an eGFR decline ≥10 ml/min/1.73m^2^. None of the investigated parameters were associated with persistent hypertension (Table 5).

**Table 2:** Baseline characteristics (median (IQR)) of women with hypertension at follow-up

	No HT (*n* = 164)	BP ≥140/90 (*n* = 38)	*p*-value	No HT (*n* = 187)	BP ≥ 150/100 (*n* = 15)	*p*-value
Age	32 (28–36)	34 (30–37)	0.16	32 (28–36)	32 (29–40)	0.59
Onset (d)	260 (241–274)	250 (233–267)	0.08	259 (241–273)	250 (217–263)	0.12
Early onset	20.3% (32/158)	29.7% (11/37)	0.21	21.1% (38/180)	33.3% (5/15)	0.27
Nulliparous	75.6% (124/162)	68% (26/38)	0.30	75.1% (139/185)	73.3% (11/15)	0.88
Time to f/u (d)	180 (155–191)	182 (132–190)	0.33	180 (153–191)	176 (121–187)	0.18
MP	14.0% (23/164)	10.5% (4/38)	0.57	14.4% (27/187)	0% (0/15)	0.11
IVF	6.7% (11/164)	5.7% (2/38)	0.74	7.0% (13/187)	0% (0/15)	0.29
GD	7.9% (13/164)	15.8% (6/38)	0.13	9.1% (17/187)	13.3% (2/15)	0.56
HELLP	21.9% (35/160)	15.8% (6/38)	0.40	21.9% 40/183	6.7% (1/15)	0.16
Severe PE	67.3% (107/159)	64.1% (25/39)	0.86	67.0% (122/182)	66.7% (10/15)	0.97
AKI	9.7% (14/145)	5.3% (3/38)	0.86	7.7% (14/182)	20.0% 3/15	0.10

*HT* Hypertension, *BP* Blood pressure in mmHg, *PE* Preeclampsia, *AKI* Acute kidney injury, *GD* gestational diabetes; *f/u* follow-up, *d* days

**Table 3:** Baseline characteristics (median (IQR)) of women with proteinuria at follow-up

	No UPE (*n* = 134)	UPE > 0.11 (*n* = 66)	*p*-value	No UPE (*n* = 184)	UPE ≥30 (*n* = 16)	*p*-value
Age	31 (28–35)	35 (28–38)	0.16	32 (29–36)	34 (25–38)	0.80
Onset (d)	260 (238–272)	258 (246–272)	0.61	259 (240–272)	266 (248–279)	0.29
Early onset	24.8% (32/129)	14.1% (9/64)	0.09	22.6% (40/177)	6.25% (1/16)	0.13
Nulliparous	75.2% (100/133)	73.9% (48/65)	0.84	73.6% (134/182)	87.5% (14/16)	0.22
Time to f/u (d)	183 (157–191)	172 (138–187)	0.06	180 (154–190)	160 (114–195)	0.42
MP	12.7% (17/134)	15.2% (10/66)	0.61	14.1% (26/184)	6.3% (1/16)	0.34
IVF	4.5% (6/134)	10.6% (7/66)	0.10	6.5%(12/184)	6.3% (1/16)	0.97
GD	7.5% (10/134)	16.7% (9/66)	0.16	8.2%(15/184)	25.0% (4/16)	0.02
HELLP	22.7% (30/132)	16.9% (11/65)	0.35	22.1% (40/181)	6.3% (1/16)	0.13
Severe	67.2% (88/131)	66.2%(43/65)	0.89	66.1% (119/180)	75% (12/16)	0.45
AKI	9.9% (13/131)	6.2%(4/65)	0.34	8.3% (15/180)	12.5% (2/16)	0.57

*UPE* urinary protein excretion, *HT* Hypertension, *BP* Blood pressure in mmHg, *PE* Preeclampsia, *AKI* Acute kidney injury, *GD* gestational diabetes; *f/u* follow-up; *d* days

**Table 4:** Baseline characteristics (median (IQR)) of women with decline in eGFR ≥10 ml/min/1.73 m2 at follow-up

	No eGFR decline (*n* = 170)	eGFR decline (*n* = 27)	*p*-value
Age	33 (28–37)	31 (27–35)	0.20
Onset (d)	258 (240–272)	261 (241–273)	0.64
Early onset	21.8% (36/165)	18.5% (5/27)	0.70
Nulliparous	75.7% (128/169)	66.7% (18/27)	0.32
Time to f/u (d)	180 (152–190)	179 (154–190)	0.93
MD	15.3% (26/170)	3.7% (1/27)	0.10
IVF	7.7% (13/170)	0% (0/27)	0.14
GD	8.8% (15/170)	14.8% (4/27)	0.33
HELLP	21.2% (36/170)	18.5% (5/27)	0.75
Severe PE	70% (119/170)	48.2% (13/27)	0.03
AKI	10.1% (17/169)	0% (0/27)	0.09

*HT* Hypertension, *BP* Blood pressure in mmHg, *PE* Preeclampsia, *AKI* Acute kidney injury, *GD* gestational diabetes, *f/u* follow-up; *d* days

**Table 5:** Multivariate logistic regression analysis for hypertension, proteinuria and eGFR at follow-up

	Odds ratio	p-value	95% conf. interval
BP ≥140/90 mmHg			
Age	1.05	0.18	0.98–1.12
Time to f/u (d)	0.99	0.08	0.98–1.00
Onset (d)	0.99	0.20	0.99–1.00
GD	2.16	0.16	0.74–6.34
		0.06	
BP ≥ 150/100 mmHg			
Time to f/u (d)	0.99	0.20	0.98–1.00
Onset (d)	0.99	0.12	0.98–1.00
HELLP	0.24	0.18	0.03–1.96
AKI	2.7	0.17	0.65–11.23
		0.08	
UPE > 11 mg/mmol			
Age	1.06	0.03	1.01–1.13
GD	1.71	0.21	0.70–4.81
Early onset	0.46	0.09	0.21–1.11
		0.02	
UPE ≥ 30 mg/mmol			
GD	3.67	0.049	1.01–13.37
HELLP	0.27	0.21	0.03–2.13
Early onset	0.35	0.45	0.02–5.46
Onset (d)	1.01	0.77	0.97–1.04
		0.08	
eGFR decline			
Severe PE	0.40	0.03	0.17–0.91

*UPE* urinary protein excretion, *BP* Blood pressure in mmHg, *PE* Preeclampsia, *AKI* Acute kidney injury, *GD* gestational diabetes *f/u* follow-up, *d* days

## Discussion

Persistent proteinuria and hypertension after preeclampsia have been reported in several studies. Proteinuria in the first few months after preeclampsia is common [11, 16, 17], and can be detected up to several years. Hypertension can also persist for months and even for years [11, 12, 18]. Decreased kidney function is uncommon even after short term follow up in contrast to persistent hypertension and proteinuria [4, 12, 16, 19]. However, there is data indicating that the risk of chronic kidney disease after preeclampsia might be increased later in life [2–4]. To our knowledge, our study is the largest on short term sequelae after preeclampsia. It shows that a significant part of patients with preeclampsia have ongoing sequelae 6 months after delivery. 20% remain hypertensive and one third have persistent proteinuria. 8% have combined hypertension and proteinuria. If hypertension or proteinuria persists 6 months after delivery, different guidelines state that referral for internal medicine or nephrology and further diagnostics should be considered [9, 10].

Berks et al. [11] reported proteinuria in 14% of patients after 3 months and 8% after 2 years. When applying, a proteinuria cut off of 0.3 g/d as done by this group, proteinuria was present in 8% in our study after 6 months, which is consistent with the 14% after 3 months in Berks’ study. However, Berks et al. measured proteinuria by 24-h urine collection, whereas a creatinine-protein ratio to estimate proteinuria was used in this study, which makes a direct comparison of this low range proteinuria results more difficult. In a study by Chua et al. [20], proteinuria was absent after 3 months, but a relatively high cut off of 0.5 g/24 h was used. Again, proteinuria was measured with 24-h urine collection. Another study reported persistent Microalbuminuria in up to 60% of patients 2–4 months after preeclampsia and in still 40% after 3–5 years using a considerably low cut off for microalbuminuria of 14 mg/24 h [19]. Overall, despite conflicting evidence proteinuria seems to be a relevant short-term sequela after preeclampsia. In our secondary analysis, older age at baseline was associated with proteinuria > 11 mg/mmol, but not with proteinuria > 30 mg/mmol. Gestational diabetes was associated with proteinuria > 30 mg/mmol. However, given the large confidence interval with the lower range close to 1, this finding might be significant by chance. Furthermore, as we know from diabetic kidney disease, it usually takes years of abnormal glucose metabolism to result in kidney damage with proteinuria, making this finding also clinically unlikely.

In our study, 20% of women were hypertensive after 6 months. In the study by Berks et al., 39% of women had hypertensive blood pressure values after 3 months and 18% were still hypertensive after 2 years [11]. These findings suggest that there is no further resolution of hypertension after 6 months in contrast to proteinuria which was only present in 2% after 2 years. Another explanation might be the difference in severe preeclampsia of 67% in our study to 89% in the study by Berks et al. and thereby a faster resolution of symptoms in our study. In another study from Japan [16] 17% (9/52) of women with preeclampsia still had hypertensive blood pressure values or received antihypertensive medication after 2 years. This value is close to the 18% of women with hypertension after 2 years in the study by Berks et al., although the percentage of patients with severe preeclampsia was not reported in the Japanese study. In a third study from Iran [12], 29% (10/35) were still hypertensive after a median follow up of 5.7 years. We could not identify any clinical parameters associated with persistent hypertension in our secondary analysis. In a subgroup analysis in 129 women with available data on peak hypertensive values at baseline, peak systolic blood pressure was significantly associated (*p* = 0.04) with persistent hypertensive values ≥ 150/100 mmHg at follow-up, but not persistent proteinuria or eGFR decline.

Data on kidney function in the first months to years after preeclampsia are limited. In the study by Shahbazian et al. [12] the mean estimated glomerular filtration rate (eGFR) after a mean follow up of 5.7 years was 108 ml/min (+/− 14 ml/min). Bar et al. found a mean creatinine of 79.2 μmol/l and 70.4 μmol/l after 2 to 4 months and after 3 to 5 years, respectively [19]. Shammas and others described no difference in renal function 10 years after hypertensive disorder in pregnancy in comparison to healthy women [17]. In our study, the mean eGFR at 6-months follow up was 110.7 ml/min/1.73m^2^. Only one woman had an eGFR below 60 ml/min/1.73m^2^. Thus, a reduced eGFR early after preeclampsia is uncommon. Our results correspond well with an analysis from Vikse et al. that showed a 0.1% risk of ESRD after preeclampsia after 30 years [4]. The improbability of chronic structural kidney disease is underlined by the absence of pathologic urine sediment findings in our study.

We arbitrarily defined kidney decline as a decrease in kidney function by ≥10 ml/min/1.73m^2^, which was present in 27 women. The clinical significance of this decline is difficult to interpret. Since physiologic glomerular hyperfiltration is still present at the end of pregnancy, the reported “decline” could really be a return to baseline before pregnancy. On the other hand, it was only present in a minority of patients and significantly associated with severe preeclampsia, both arguments against a physiological process. However, although a signifcant difference (*p* < 0.00) in medians in eGFR in women with a decline in eGFR (103 ml/min/1.73m^2^) and women without (114 ml/min/1.73m^2^) was present, both were within normal range. Furthermore, as mentioned above the risk of ESRD after preeclampsia is far lower than the 13% (27/197) of women with an eGFR delcine in our study, thus the clinical significance remains unclear.

Our study has several strengths. To the best of our knowledge, this is the largest study on short term sequelae of preeclampsia. Furthermore, information on urine sediments has not been reported in this setting before. Due to a standardized protocol for follow-up after preeclampsia at our clinic, a low number of patients were lost to follow-up. The collection of several baseline characteristics allowed for secondary analysis for risk factors.

There are also several limitations. Being designed as a cohort study, there is no control group as a reference. We did not have information on GFR during pregnancies, since there were no routine blood tests before preeclampsia symptoms occurred with mostly initiation of delivery short thereafter. An analysis on possible sociodemographic differences between the patients lost to follow-up and the patients included in our study would have been of interest, but was not possible due to unavailability of sociodemographic data. There seems to be a selection bias with a high incidence for severe preeclampsia (67%), most likely due to our hospital being a tertiary center with referral of more complicated deliveries. Additionally, even though all patients with preeclampsia in our center are supposed to be referred for nephrology follow-up after 6 months, we cannot exclude that some patients with mild forms of preeclampsia were not referred for follow-up leading to a higher incidence of severe preeclampsia.

## Conclusion

Given the high frequency of sequelae 6 months after preeclampsia, this study underlies the importance of close follow-up. To identify those women at risk for persistent symptoms, knowing what features of preeclampsia are associated with these short-term sequelae would be important. We identified age and gestational diabetes as risk factors for proteinuria and severe preeclampsia for a decline in eGFR. However, further studies are need to define the clinical relevance of these findings.

## Abbreviations

CKD-EPI: Chronic kidney disease epidemiology collaboration
eGFR: Estimated glomerular filtration rate
ESRD: End-Stage Renal Disease
HELLP: Hemolysis, Elevated Liver Enzymes, Low Platelet
SD: Standard Deviation
